# Progress on mechanisms of age-related hearing loss

**DOI:** 10.3389/fnins.2023.1253574

**Published:** 2023-08-30

**Authors:** Wen Yang, Xiaolong Zhao, Renjie Chai, Jiangang Fan

**Affiliations:** ^1^Department of Otolaryngology Head and Neck Surgery, Sichuan Provincial People's Hospital, University of Electronic Science and Technology of China, Chengdu, China; ^2^State Key Laboratory of Bioelectronics, Department of Otolaryngology Head and Neck Surgery, Zhongda Hospital, School of Life Sciences and Technology, Advanced Institute for Life and Health, Jiangsu Province High-Tech Key Laboratory for Bio-Medical Research, Southeast University, Nanjing, China; ^3^Co-Innovation Center of Neuroregeneration, Nantong University, Nantong, China

**Keywords:** age-related hearing loss, presbycusis, oxidative stress, mitochondrial DNA damage, inflammation

## Abstract

Age-related hearing loss, or presbycusis, is a common cause of hearing loss in elderly people worldwide. It typically presents as progressive, irreversible, and usually affects the high frequencies of hearing, with a tremendous impact on the quality of life. Presbycusis is a complex multidimensional disorder, in addition to aging, multiple factors including exposure to noise, or ototoxic agents, genetic susceptibility, metabolic diseases and lifestyle can influence the onset and severity of presbycusis. With the aging of the body, its ability to clean up deleterious substances produced in the metabolic process is weakened, and the self-protection and repair function of the body is reduced, which in turn leads to irreversible damage to the cochlear tissue, resulting in the occurrence of presbycusis. Presently, oxidative stress (OS), mitochondrial DNA damage, low-grade inflammation, decreased immune function and stem cell depletion have been demonstrated to play a critical role in developing presbycusis. The purpose of this review is to illuminate the various mechanisms underlying this age-related hearing loss, with the goal of advancing our understanding, prevention, and treatment of presbycusis.

## Introduction

Age-related hearing loss (ARHL), or presbycusis, is the most common sensory deficit affecting aging adults (Agrawal et al., 2008). ARHL typically presents as a progressive, irreversible sensorineural hearing loss that increases with age, mainly involving high frequency hearing, and gradually spreads to low frequency hearing (Wang and Puel, 2020). Presbycusis is not only associated with damage to the cochlear organs but is often accompanied by central nervous system dysfunction that reduces the ability to process auditory information (Basner et al., 2014). Therefore, while auditory sensitivity decreased, patients may also experience reduced understanding of speech in noisy environments, as well as slowed central processing of acoustic information and impaired localization of sound sources (Boettcher, 2002; Bielefeld et al., 2010; Fetoni et al., 2011).

According to a 2012 estimate by the World Health Organization, about 164.5 million people over the age of 65 suffer from hearing impairment worldwide, accounting for about 33% worldwide in adults older than 65 years (Sheffield and Smith, 2019). The prevalence of hearing loss increases with age, and it can reach 84.3% among people over 80 years old (Yamasoba et al., 2013). With the aggravation of population aging and the extension of life expectancy, it is estimated that more than 500 million individuals over the age of 60 will affected by presbycusis by 2025 (Wang and Puel, 2020). Presbycusis often presents with communication difficulties, reduced quality of life, and social isolation (Kay et al., 1964). In recent years, some studies have shown that presbycusis can lead to cognitive decline and is an independent risk factor for a series of neuropsychiatric diseases such as depression, dementia and Alzheimer’s disease (Lin et al., 2011, 2013). Those creates a large socioeconomic burden.

Presbycusis is a complex chronic aging disease that results from the gradual accumulation of deleterious biological lesions and auditory system dysfunction (Eckert et al., 2021). With the aging of the body, a series of changes occur in the auditory organs. For example, the irreversible degeneration of the cochlear stria vascularis, spiral ligament, hair cells, and auditory nerve fibers can lead to blockage of ion and signal transmission, the exhaustion of stem cells weakens the regenerative repair ability of fibroblasts in the spiral ligament, and the decline of immune function leads to persistence of chronic inflammation, reduced enzyme activity can weaken the body’s ability to remove toxic substances and ultimately leading to hearing loss (Seidman et al., 2002; Lang et al., 2006; Watson et al., 2017; Tawfik et al., 2020; Eckert et al., 2021). However, the age of onset and the degree of hearing loss vary greatly among individuals. Presbycusis is influenced by multiple factors and represent the interaction of numerous intrinsic and extrinsic factors. In addition to aging, many factors such as exposure to noise, or ototoxic agents, genetic susceptibility, metabolic diseases and lifestyle can all contribute to the development of presbycusis alone or in combination (Keithley, 2020; Wang and Puel, 2020). Those factors mainly lead to the occurrence of hearing loss through the mechanism of oxidative stress (OS), mitochondrial DNA damage, low-grade inflammation and reduced vascularisation in the cochlea (Seidman et al., 2002; Watson et al., 2017). The purpose of this review is to illuminate the pathophysiological features, etiology and pathogenesis of presbycusis, with the goal of advancing our understanding, prevention, and treatment of presbycusis.

### Pathophysiological features of presbycusis

The cochlea is a spiral-shaped cavity composed of three liquid-filled compartments, scala media, scala tympani and scala vestibuli, which can convert the mechanical vibration caused by sound waves into electrical signals during sound transmission and is an important target organ for presbycusis. Scala media contains endolymph with high potassium and low sodium and is sandwiched by scala tympani and scala vestibuli containing perilymph with low potassium and high sodium. The endolymph in the scala media contains a positive voltage of +80 to +100 mV (endocochlear potential: EP), which is generated by the potential difference between the endolymph in scala media and the perilymph in scala tympani (Hibino et al., 2010; Bazard et al., 2021a). The stria vascularis and spiral ligaments located on the cochlear lateral wall have an intact ion channel transport system that transports potassium ions into the endolymph to maintain the high potassium status of the endolymph and the highly positive EP, which is essential for hair cell transmission (Wangemann, 2006).

Auditory information transmission is a complex process, and the cochlea is an important organ in this process. The shear motion caused by acoustic vibration causes the cilia of hair cells to bend or deflect, which in turn opens potassium channels at the top of hair cells, allowing potassium ions in the endolymph to flow into the hair cells to produce depolarization. Depolarization of hair cells causes calcium channels to open in cells and calcium ions to influx, which in turn prompts hair cells to release neurotransmitters into the synaptic cleft of the hair cells and auditory neurons (Bazard et al., 2021a). Damage to cells and tissues associated with auditory information conduction, as well as changes in the cochlear microenvironment, may contribute to the development of presbycusis (Noble et al., 2022).

According to postmortem histopathological studies, a variety of pathological changes occur in the inner ear of patients with presbycusis, such as the atrophy of the stria vascularis (SV) and loss of fibrocytes of the spiral ligament (SL) in the lateral wall of the cochlea (strial presbycusis, also known as metabolic presbycusis), decrease of sensory hair cells (sensory presbycusis) and degeneration of the auditory nerve (neural presbycusis) (Ohlemiller, 2004; Bowl and Dawson, 2019). The cochlear lateral wall degeneration is an important pathological change in in aging. Even the quiet-aged gerbil that raised under strictly controlled experimental conditions will experience degeneration of the stria vascularis and spiral ligament at both ends of the cochlear duct with aging (Spicer and Schulte, 2002). Deterioration of the lateral wall of the cochlea reduces the number and function of the sodium-potassium pump (Na-K-2Cl cotransporter NKCC1 and Na+, K + -ATPase), resulting in impaired potassium circulation and reduced EP (Chen and Zhao, 2014; Bazard et al., 2021a). Hair cells (HCs), which transduce mechanical stimuli into electrical activity through the hair bundle on their apical surface, degenerate with age and are also susceptible to factors such as noise exposure and ototoxic drugs (Bazard et al., 2021a). HCs damage is predominantly outer hair cells (OHCs), and it begins both apical and basal ends of the cochlea and progresses throughout the length of the organ of Corti, while inner hair cells (IHCs) damage is less and restricted to the extreme basal end of the aging cochlea (Kujawa and Liberman, 2019). The EP and OHCs have the function of cochlear amplification, which can provide 50–70 dB of gain in the basal turn of the cochlea (high-frequency hearing threshold region), while the apical of the cochlea (low-frequency hearing threshold region) can only gain 20 dB (Lang et al., 2010). This may explain the greater impairment of high-frequency hearing thresholds when the cochlear lateral wall and OHCs are damaged. IHCs are sensory receptors that transmit amplified information to the brain via spiral ganglion neurons and auditory nerve. Aging or noise-accumulating lesions can lead to degenerative changes in spiral ganglion neurons (SGNs), in which low-spontaneous rate (SR) fibers are more susceptible to damage and lesions usually involve both apical and basal ends of the cochlea (Lang et al., 2010). The low-SR fibers mainly contribute to encoding transient stimuli in the background of noise, while not threshold detection in quiet situations (Furman et al., 2013). Therefore, neural presbycusis is mainly manifested as reduced understanding of speech in noisy environments, while the hearing threshold nearly normal in quiet. With aging, the cochlear vascular also exhibits pathological changes, such as merged capillaries, reduced red blood cell velocity and vascular plasticity, and thickened basement membrane, resulting in weakened oxygen and nutrient delivery and waste elimination (Ohlemiller et al., 2008; Eckert et al., 2021). Secondly, the permeability of the strial microvasculature increases, allowing harmful substances to enter the cochlea (Seidman et al., 1996).

However, in reality, affected by various pathogenic factors, presbycusis can cause lesions in multiple parts of the cochlea, showing a “mixed” pathology (Tawfik et al., 2020). In addition, presbycusis may have no obvious histopathological changes under light microscopy, but submicroscopic structural changes may occur, such as stereociliary lesions or reduced synapses between inner hair cells and afferent fibers (Sliwinska-Kowalska and Davis, 2012). Liu et al. found that aging mouse had loss of stereocilia and shrinkage of hair cell soma precede hair cell loss. After acoustic overstimulation, synaptic connections also disappear before hair cells in presbycusis (Liu et al., 2022).

### Risk factors of presbycusis

#### Genetic susceptibility

Presbycusis is a disease with genetic susceptibility. According to twins studies and longitudinal studies of family cohorts, its heritability is small to moderate, with a heritability indices of between 0.35 and 0.55 (Bowl and Dawson, 2019). Unlike the single-gene genetic pathogenic pattern of congenital deafness and early-onset deafness, it is generally believed that presbycusis involves multiple genetic variants, each of which has a small impact (Wells et al., 2020).

Genome-wide association study (GWAS) is widely used for genetic composition analysis of presbycusis, and many candidate genes have been found to be associated with presbycusis (Bowl and Dawson, 2019). These genes may play an important role in signaling and maintenance of the cochlear microenvironment. Several independent studies have reported that the gene encoding glutamate metabotropic receptor 7 (GRM7) is associated with presbycusis. Mutations in *GRM7* may lead to the accumulation of neurotransmitters in synaptic connections, thereby altering the susceptibility to presbycusis (Van Laer et al., 2010; Newman et al., 2012). Genetic polymorphisms in the genes coding detoxification enzymes are also linked to presbycusis, such as Uncoupling protein 2 (*UCP2*), Superoxide dismutase 2 (*SOD2*) and N-acetyltransferase 2 (*NAT2*) (Arsenijevic et al., 2000; Unal et al., 2005). Additionally, genetic variation may also contribute to increased susceptibility to presbycusis by environmental factors such as noise and ototoxic drugs (Prezant et al., 1993; Sliwinska-Kowalska and Pawelczyk, 2013).

Monogenic deafness-causing genes, especially those that cause delayed-onset deafness, may also be associated with presbycusis. Wells et al. (2019) performed GWAS for self-reported hearing loss adults in UK Biobank and reported 10 of the 44 associated loci included monogenic deafness genes. *TMC1* variant was previously thought to be associated with progressive postlingual hearing loss and profound prelingual deafness. However, in a recent study, Boucher et al. (2020) demonstrated through *in vitro* transfection and *in vivo* animal models that the heterozygous pathogenic variants of *TMC1* can cause presbycusis in a single-gene form, which updates our understanding of the inheritance pattern of presbycusis and provides a basis for potential inner ear treatments.

#### Noise exposure

Noise is the second most common cause of hearing loss other than old age, and noise-induced hearing loss (NIHL) is considered a common occupational disease, with a high incidence in occupational workers who have been exposed to noise for a long time, such as workers in textile, mining, and heavy engineering industries (Nandi and Dhatrak, 2008; Natarajan et al., 2023). Both aging and acoustic trauma can lead to loss of hair cells at the base end of the cochlea. Aged animals raised in quiet environments show did not lose hair cells until well past the middle of the lifespan, and the loss was small, whereas human temporal bone specimens have found stable and large loss of hair cells throughout the life (Kujawa and Liberman, 2019). This suggest that noise exposure synergizes with aging in the development of presbycusis.

Noise damage to the auditory system is affected by intensity and duration of noise exposure and can cause permanent threshold shifts (PTS) or temporary threshold shift (TTS) (Natarajan et al., 2023). Both long-term high-intensity noise exposure and one-time exposure to hazardous noise levels can lead to PTS (Liberman, 2016; Ryan et al., 2016). Due to the repair function of the stereocilia tip links, moderate noise damage often leads to temporary hearing loss, which recovers within 24–48 h (Gerhardt et al., 1987; Jia et al., 2009). Although low-intensity noise stimulation does not directly cause hair cell loss, it may causes permanent damage to the stereocilia bundles on the hair cells and to the synaptic connections between the auditory nerve fibers (ANF) and the IHC, which results in a blockage of auditory signal transmission and decreased ability to distinguish speech in noisy background (Kujawa and Liberman, 2019). Therefore, noise exposure has a cumulative effect on damage to the auditory system, prolonged exposure to 70 dB of noise may also cause hearing damage, and long-term lower noise and short-term louder noise have the same effect on hearing (Natarajan et al., 2023).

#### Ototoxic agents

Aminoglycoside antibiotics and chemotherapy drugs such as cisplatin and carboplatin can cause degenerative changes in the cochlea and hearing loss (Jiang et al., 2017). Additionally, through a large longitudinal cohort study lasting 10 years, Joo et al. (2020) found that loop diuretics and nonsteroidal anti-inflammatory drugs were associated with risk of progressive hearing loss, and may contributor to the incidence and severity of age-related hearing loss.

#### Metabolic diseases

Metabolic diseases are a cluster of diseases or disorders that disrupt normal metabolism, including high blood sugar (hyperglycemia), increased blood pressure (hypertension), excess fat around the waist (obesity), and abnormal levels of cholesterol or triglycerides (dyslipidemia). In recent years, under the influence of unhealthy diet and lifestyle, the incidence of metabolic diseases and its components are on the rise, and it is most common in the elderly (Saklayen, 2018). Presbycusis and metabolic diseases are both chronic diseases with a high prevalence, and many older people suffer from them at the same time (Guo et al., 2022). In a large cohort study of 94,223 people in Korea, Rim et al. (2021) reported that obesity, hypertension, hyperglycemia and dyslipidemia were all strongly associated with hearing loss, and the number of components of the metabolic diseases is positively correlated with the rate of sensorineural hearing loss. In two cohort studies in Europe and Korea, high body mass index (BMI) and low BMI were found to be associated with hearing loss, respectively (Fransen et al., 2008; Lee et al., 2015). Furthermore, Nguyen et al. (2022) found that the mouse model of diabetes and dyslipidemia had higher hearing impairment and degeneration of the cochlear spiral ganglion and stria vascularis. Some studies have found mitochondrial dysfunction occurs in both metabolic diseases and presbycusis, and Guo et al. (2022) suggested that metabolic diseases may increase susceptibility to presbycusis by causing mitochondrial dysfunction.

#### Lifestyle

Lifestyle effects on hearing are diverse, with studies showing that both smoking and passive smoking increase the risk of hearing loss, while moderate alcohol consumption has a protective effect on hearing (Dawes et al., 2014). Diet and exercise may also play a role in aging and hearing. A high antioxidant diet can reduce mitochondrial dysfunction, thereby decreasing the magnitude of the vascular atrophy and cochlear auditory nerve degeneration (Le and Keithley, 2007). Han et al. (2016) found that increasing exercise in mice was effective in attenuating cochlear degeneration and hearing loss.

### Age-related changes and pathogenesis of presbycusis

#### Age-related reactive oxygen species accumulation and mitochondrial DNA damage

Reactive oxygen species (ROS) are highly reactive chemicals produced during mitochondrial respiration or cellular response to endogenous and exogenous factors, mainly including superoxide anion (O2-), hydrogen peroxide (H2O2), hydroxyl radical (OH-) and nitric oxide (NO-) (Pizzino et al., 2017). ROS can serve as critical signaling molecules in cell proliferation and survival, but their excessive production and accumulation can lead to oxidative stress (OS), which in turn leads to macromolecular damage, promoting diseases such as presbycusis, aging and cancer (Ray et al., 2012). In the cochlea, ROS can damage DNA, break down lipid and protein molecules, and lead to cochlear cell apoptosis (Paplou et al., 2021). Increased plasma levels of ROS in humans are associated with hearing loss, while a high antioxidant diet can reduce cochlear degeneration and hearing loss (Le and Keithley, 2007; Lasisi and Fehintola, 2011). In fact, there are antioxidant enzymes in the body that can remove ROS, such as glutathione reductase (GSR), superoxide dismutase (SOD), catalase (CAT) and methionine sulfoxide reductase (MSR). The balance of the body’s antioxidant enzymes and ROS can avoid damage to cells and tissues caused by OS (Seidman et al., 2002; Paplou et al., 2021).

The cochlea is an energy-intensive organ in which mitochondria provide energy for their sodium-potassium pump activity and ion transport through oxidative phosphorylation, while producing large quantities of ROS. The aggregation of ROS in the cochlea can lead to mutations in the mitochondrial genome, resulting in mitochondrial DNA (mtDNA) damage (Seidman et al., 2002). Mitochondrial DNA mutation is an important component of auditory system damage, and its characteristic 4,977 bp deletion occurs frequently in temporal bone tissue samples from patients with presbycusis (Zhong et al., 2011). In addition, postmortem analysis of the temporal bones of patients with presbycusis revealed defects in the expression of mitochondrial aerobic metabolism-related enzymes (Markaryan et al., 2009). For the damaged mitochondria, cells can clear and renew them through the autophagy and mitochondrial dynamics (fission and fusion events) (Wang and Puel, 2018).

**Figure 1:**
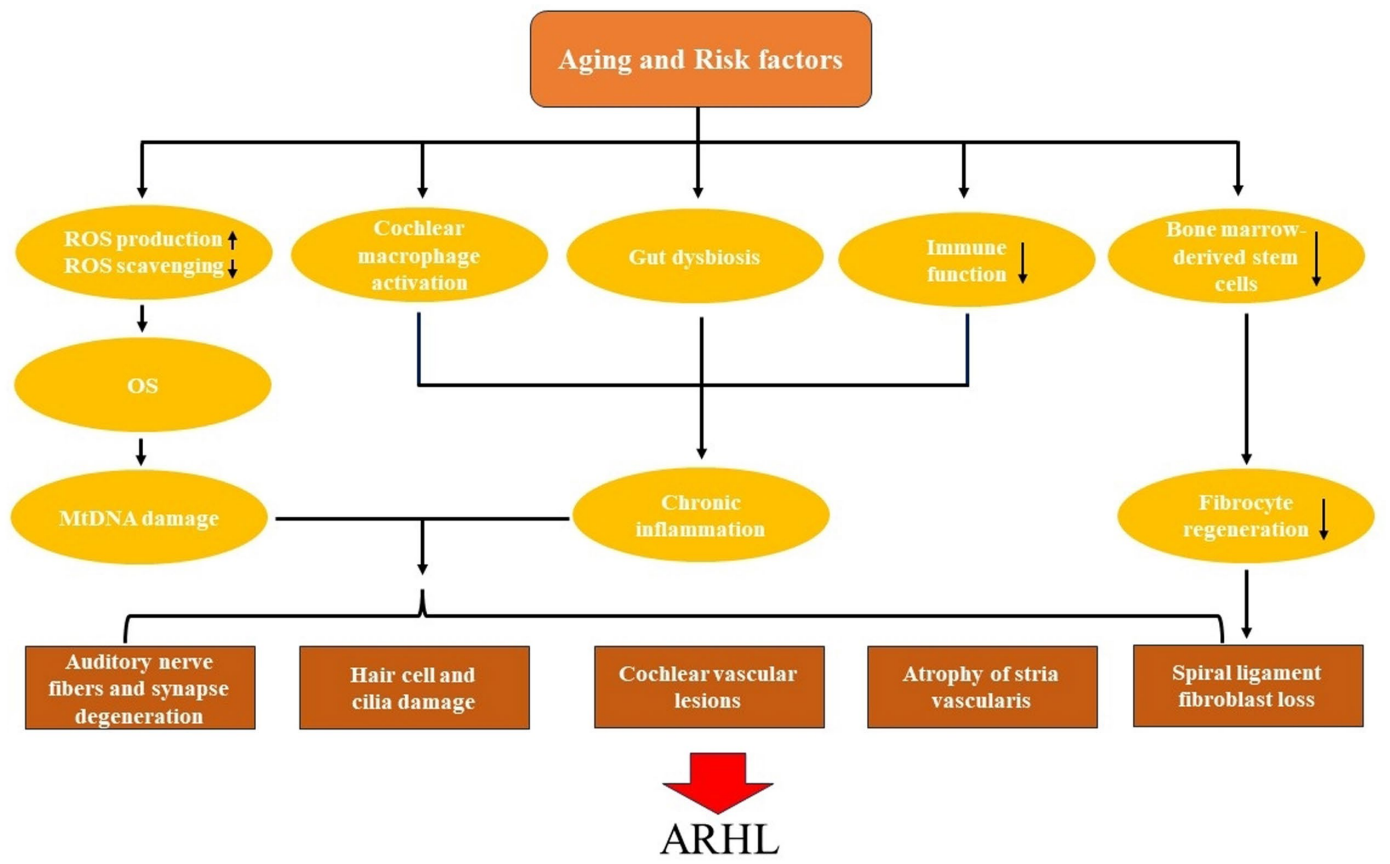
The pathogenesis and pathological changes of age-related hearing loss (ARHL).

However, as the body ages, the production and function of antioxidant enzymes decrease, and ischemic and hypoxic damage from local vascular lesions in the cochlea leads to increased production of ROS (Ray et al., 2012). As a result, the original balance is broken, and OS will cause cumulative damage to mitochondria and cochlear cells. In addition, mitochondrial biogenesis in the elderly is weakened, autophagy and mitochondrial dynamics are reduced, so that mitochondria are constantly depleted, so that normal cells and cochlear function cannot be maintained (Seidman et al., 2002; Wang and Puel, 2020; Figure 1).

#### Inflammaging and decreased immune function

Inflammaging is chronic and low-grade inflammation of tissues and organs that occurs during aging and can lead to conditions as diverse as cardiovascular disease, diabetes, and neurodegenerative diseases (Bazard et al., 2021b; Kociszewska and Vlajkovic, 2022). Various stimuli, including cellular debris, nutrients and pathogens, can drive sterile inflammation (Franceschi et al., 2018). The body’s immune function declines with age, mainly manifested by shift in T-cell subpopulation distribution (the number of naive T cells (especially CD8+) decreases and homeostatically proliferate into memory T cells), impaired calcium-mediated signaling and thymic atrophy, resulting in weakened immune surveillance and clearance of pathogens (Goronzy and Weyand, 2013; Yousefzadeh et al., 2021). In turn, it leads to the accumulation of inflammatory stimuli, which continuously stimulates the body to produce chronic inflammatory responses. The cochlea is not an immune-privileged organ. Systemic inflammation is associated with presbycusis, and changes in the morphology and number of macrophages can occur in the aging cochlea (Watson et al., 2017).

Macrophages are a key part of the innate immune system, presented in the cochlear spiral ligament, the auditory nerve, and the organ of Corti. Activated macrophages transform from highly branched morphology to amoeboid shape and can phagocytose cellular debris or pathogens, which are critical for maintaining the homeostasis of the cochlear microenvironment (Noble et al., 2022). In addition, the vascularization function of macrophages can regulate the permeability of the blood-labyrinth barrier (BLB) of the strial microvasculature (Noble et al., 2022). Noise trauma activates macrophages, causing changes in their shape and number (Presta et al., 2018). Analysis of temporal bone specimens of different ages revealed that aging cochlear macrophages were highly activated (Noble et al., 2019). The inflammatory response driven by macrophage activation plays an important role in the development of presbycusis by leading to cochlear degeneration and increased stria vascular permeability (Noble et al., 2022).

The intestinal tract contains a large number of microorganisms and their genetic material, and gut dysbiosis may also contribute to inflammaging. In addition to the high-fat diet can lead to the development of cochlear inflammation (Kociszewska et al., 2021). As the body ages, a series of changes occur in the intestinal tract, such as the reduced microbiota diversity, more pro-inflammatory microbiota such as LPS-producing Gram-negative bacteria, and the permeability of the intestinal barriers increases (Ragonnaud and Biragyn, 2021). Therefore, pathogens and metabolites of intestinal microorganisms can be transported to the cochlea through the systemic circulation, leading to chronic inflammation of the cochlea and the occurrence of presbycusis (Kociszewska and Vlajkovic, 2022; Figure 1).

#### Fibrocyte regeneration and stem cell depletion

The spiral ligament is a component of the potassium ion transport system in the lateral wall of the cochlea, which contains five types of fibrocytes, and its normal function is crucial for the maintenance of the EP (Spicer and Schulte, 1991; Lang et al., 2003; Eckert et al., 2021). Unlike sensory hair cells and neurons, which do not regenerate, spiral ligament fibrocytes typically recover rapidly after ototoxic drug and noise damage (Roberson and Rubel, 1994; Lang et al., 2003). The regenerative repair ability of fibrocytes relies on bone marrow-derived stem cells (Lang et al., 2006). However, as the body ages, stem cells are continuously depleted and their differentiation potential and proliferation rate decrease (Fehrer and Lepperdinger, 2005). Therefore, the renewal and repair ability of fibrocytes in the elderly is weakened, the spiral ligament atrophies, and metabolic presbycusis occurs (Eckert et al., 2021; Figure 1).

### Prevention and treatment of presbycusis

#### Prevention of presbycusis

The management of presbycusis should first focus on prevention and avoid exposure to risk factors. While we cannot prevent aging, nor can we change our genetic background, we can minimize noise exposure, wear earplugs in noisy environments, and avoid ototoxic medications (He et al., 2019). Elderly patients should actively treat metabolic diseases and ear infections to avoid damage to their hearing (Guo et al., 2022). A good lifestyle is also essential for the prevention of presbycusis, and a reduced intake of fatty foods and a diet high in antioxidants can reduce hearing loss (Le and Keithley, 2007; Kociszewska et al., 2021). In addition, proper exercise not only strengthens immune function, but also reduces free radicals in the body. Studies have shown that long-term exercise can delay the progression of presbycusis by reducing age-related capillary loss associated with inflammation (Han et al., 2016).

#### Treatment of presbycusis

Treatment of presbycusis still relies clinically on hearing amplification and cochlear implantation. Air conduction hearing aids are commonly worn in patients with mild to moderate hearing loss, active middle ear implants can be used in patients with moderate to severe hearing loss, and cochlear implants should be considered in patients with severe to profound hearing loss (Seidman et al., 2019). However, it is estimated that only 15% of eligible patients use them due to multiple factors including cost, appearance, discomfort, and lack of perceived benefit (Chien and Lin, 2012; Mahboubi et al., 2018).

In recent years, some researchers have begun to explore the treatment of presbycusis with antioxidants, anti-inflammatories, neurotrophins and other drugs (Wang and Puel, 2020). Studies by Benkafadar et al. (2019) show that EUK-207, a synthetic superoxide dismutase/catalase mimetic, reduced hair cell degeneration and age-related hearing loss in senescence-accelerated mouse-prone 8 (SAMP8) mice. Serra et al. (2022) believe that although the antioxidant melatonin cannot completely prevent presbycusis, it can delay its occurrence. Aspirin displays anti-inflammatory and antioxidant properties, and studies have shown that it can effectively reduce hearing loss in aged mice (Cazals, 2000). An ongoing clinical trial is attempting to assess its potential therapeutic effect on presbycusis in humans (Lowthian, et al., 2016). Cassinotti et al. (2022) overexpressed neurotrophin-3 (Ntf3) in mouse cochlea starting at middle age, thereby preventing age-related inner hair cell synaptopathy and slowing age-related hearing loss. Oral treatment with selegiline, a neuroprotective antiparkinsonian drug, significantly alleviated hearing loss at higher frequencies in mice with moderate hearing loss, but not in mice with rapid progressive hearing loss (Szepesy et al., 2021). We believe that the drug treatment of presbycusis has broad prospects, but the current application is still concentrated in animal models and clinical trials, and its clinical application still needs to solve problems such as efficacy, safety, administration route and dosage. In addition, gene therapy and stem cell transplantation also have potential treatment for presbycusis, which still need further research (Chen et al., 2012; Davidsohn et al., 2019).

## Conclusion

Presbycusis is a common chronic disease occurring in the process of aging, which is the result of the interaction of various extrinsic and intrinsic factors under the genetic background. With the aging of the body, the function or number of immune functions, antioxidant enzymes and self-repairing stem cells that are closely related to the body’s self-protection decline, resulting in the accumulation of inflammatory cytokines, reactive oxygen species and tissue cell damage in the body. Therefore, under the action of chronic inflammation and oxidative stress, irreversible damage occurs to the cochlear stria vascularis, spiral ligament, sensory hair cells and auditory nerve fibers, and then lead to the occurrence of presbycusis.

Mitigating exposure to risk factors is essential for the prevention of presbycusis. The clinical treatment of presbycusis is still mainly based on wearing hearing aids and cochlear implants, but various drugs launched according to its pathogenesis are also undergoing clinical research. The in-depth exploration of the pathogenesis of presbycusis provides a reference for potential drug treatment and clinical intervention.

## Author contributions

WY: Writing – original draft, Methodology. XZ: Writing – original draft, Methodology. RC: Visualization, Writing – review & editing. JF: Validation, Writing – review & editing.

## Conflict of interest

The authors declare that the research was conducted in the absence of any commercial or financial relationships that could be construed as a potential conflict of interest.

## Publisher’s note

All claims expressed in this article are solely those of the authors and do not necessarily represent those of their affiliated organizations, or those of the publisher, the editors and the reviewers. Any product that may be evaluated in this article, or claim that may be made by its manufacturer, is not guaranteed or endorsed by the publisher.
